# A complete chloroplast genome sequence of *Viola albida* Palibin 1899 (Violaceae), a member of VIOLA ALBIDA complex

**DOI:** 10.1080/23802359.2023.2224462

**Published:** 2023-06-18

**Authors:** Hyeonji Moon, Sangtae Kim

**Affiliations:** Department of Biology, Sungshin Women’s University, Seoul, Korea

**Keywords:** Chloroplast genome, phylogeny, *Viola albida*, VIOLA ALBIDA complex, Violaceae

## Abstract

The VIOLA ALBIDA complex is a complicated group with taxonomic problems having continuous leaf variations and composed of taxa related to the following names: *Viola albida*, *V. albida* var. *takahashii*, and *V. chaerophylloides.* As a first step to understanding the genomic nature of this complex, this study identified the whole chloroplast genome of *V. albida*. The genome is 157,692 bp in length (36.3% of GC content) and contains four subregions: a large single copy region of 86,220 bp, a small single copy region of 17,248 bp, and a pair of inverted regions of 27,112 bp each. An annotation of the gene identifies 111 unique genes, including 77 protein-coding genes, four rRNA genes, and 30 tRNA genes. The phylogenetic analysis of this genome with selected cp genomes from *Viola* identifies the close relationship between *V. albida* and *V. ulleungdoensis.* It is noteworthy that *V. chaerophylloides*, traditionally recognized as a member of the VIOLA ALBIDA complex, is genetically distant from *V. albida* and forms a sister group of all other members of the subsection *Patellares*. Our genome report is expected to serve as a basis for understanding the identity of the VIOLA ALBIDA complex.

## Introduction

*Viola* L. (Violaceae) is the 40th to 50th largest genera in angiosperms and contains ca. 664 species mainly distributed in temperate regions and highlands in tropical mountains (Marcussen et al. 2022). The type specimen of *Viola albida* Palibin (Palibin 1899) was collected in Seoul, Korea, and the species is distributed in far Eastern Russia, Northern China, and the Korean peninsula (https://powo.science.kew.org/). The species is included in subgenus *Viola,* section *Plagiostigma*, and subsection *Patellares* in the recent classification system of *Viola* (Marcussen et al. 2022). *V. albida* is distinguished from other *Viola* species in Korea by plants without stems, showy white flowers having spherical-apex style, stipules adnate to petiole, undissected leaves having shallowly serrated margins, etc. (Lee and Yoo 2020, Figure 1). As closely related taxa, *V. albida*, *V. albida* var. *takahashii* (Nakai) Nakai (Nakai 1916), and *V. chaerophylloides* (Regel) W. Becker (Becker 1902) exhibit morphological continuity in leaves: from undivided (*V. albida*) through deeply lobed (*V. albida* var. *takahashii*) to fully divided (compound leaves; *V. chaerophylloides*) (Choi and Whang 2019). Since they have a complex taxonomic history, often recognized as independent species or varieties of *V. albida*, these taxa are recognized as the VIOLA ALBIDA complex (Whang 2006; Choi and Whang 2019). Various approaches have been conducted to understand the evolution of VIOLA ALBIDA complex: leaf morphological (Kim et al. 1991; Jang et al. 2006), genetic (Ko et al. 1998; Koo et al. 2010), developmental (Choi and Whang 2019), transcriptome (Srikanth et al. 2019), and phylogenetic (Whang 2006) studies. Interestingly, two previous molecular phylogenetic studies of Korean violets based on two (Yoo et al. 2007) and eight (Yoo and Jang 2010) chloroplast (cp) DNA regions, respectively, showed that VIOLA ALBIDA complex do not form an independent clade: these studies provided insufficient phylogenetic information. Therefore, unraveling the phylogenomic structure of the VIOLA ALBIDA complex requires analyses with more data, such as analyses based on cp whole-genome data or nuclear HybSeq (Weitemier et al. 2014) data. As the first step for understanding the genome evolution of the VIOLA ALBIDA complex, we report a complete chloroplast genome sequence of *V. albida*, a typical member of the VIOLA ALBIDA complex, in this study.

**Figure 1.**
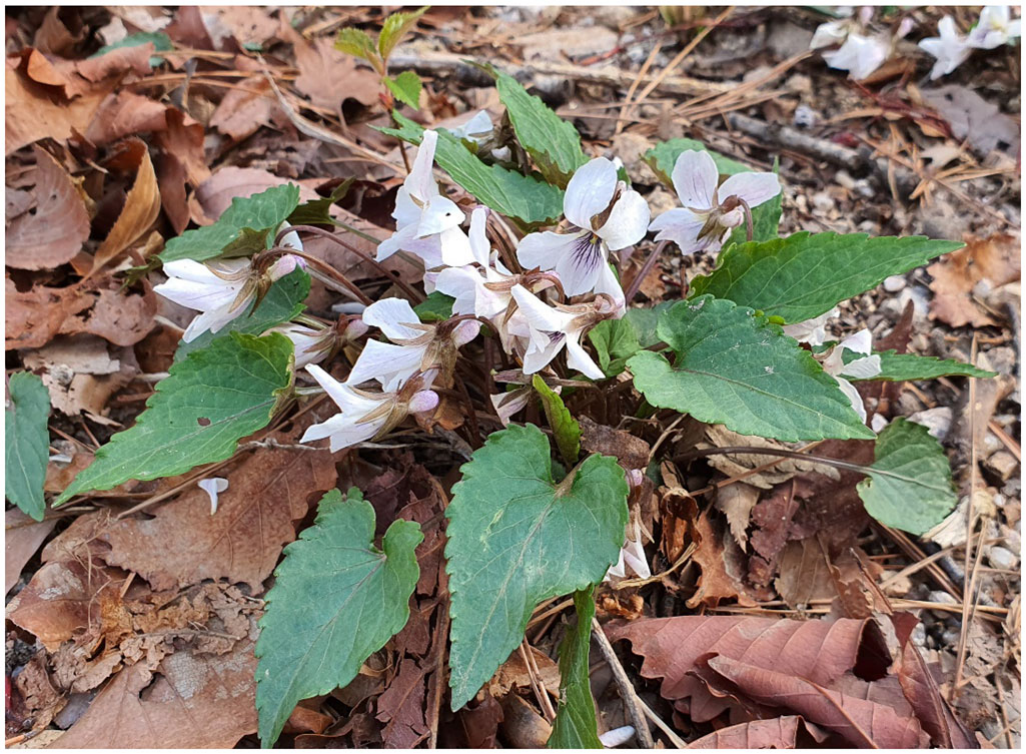
The habitat of *V. albida* distributed in Mt. Bukhan, Seoul, Korea (photo by Sangtae Kim). The species has showy white flowers and undissected leaves with shallow-serrated margins.

## Materials and methods

A sample for the study was collected from Mt. Bukhan, Seoul, Korea (N 37°37'47.04", E 126°58'35.89"). The voucher specimen (*S. Kim 2020-001,* SWU) was deposited at the herbarium of Sungshin Women’s University (SWU; Sangtae Kim, amborella@sungshin.ac.kr). With a commercial kit (Exgene^TM^ Plant SV; GeneAll Biotechnology Co. Ltd., Seoul, Korea), total genomic DNA was extracted from a fresh leaf. We produced 25,132,239 paired-end reads (150 bp) using the MGISEQ-2000 platform (MGI Tech Co. Ltd., Shenzhen, China). Following the manufacturer’s manual, a paired-end sequencing library was prepared with the MGIEasy DNA library Prep kit (MGI Tech Co. Ltd., Shenzhen, China). After the library construction, the extracted DNA was stored in the DNA Bank of the Plant Molecular Phylogeny Lab, Department of Biology, Sungshin Women’s University. We obtained 7.53 Gbps of sequence after the low-quality filtration (>Q30: 88.6%) by Trimmomatic (Bolger et al. 2014). In order to determine the cp genome of *V. albida*, the paired-end reads were mapped against the cp genome of *V. ulleungdoensis* (NC_050744), of which phylogenetic position is closely related to the members of the VIOLA ALBIDA complex (Yoo and Jang 2010; Yang et al. 2021), with the mapping module in the Geneious prime (v. 11.0.11 + 7; 'medium-low’ sensitivity option; Kearse et al. 2012). When we manually checked the entire sequence regions, the whole genome was perfectly mapped with apparent substitutions and indels, and no severe mismatching regions were detected. A consensus sequence was produced, and it was confirmed again with the mapping of raw reads against the consensus sequence for the completion of cp genome of *V. albida*. Gene annotation of the consensus sequence was performed by GeSeq (Tillich et al. 2017) with the seven previously reported *Viola* cp genomes (NC_050744.1, NC_041585.1, NC_026986.1, NC_041584.1, NC_052919.1, NC_041583.1, and NC_041582.1) as references. The annotations were manually checked and edited by the comparison with the above seven *Viola* cp genomes using Geneious prime (v. 11.0.11 + 7; Kearse et al. 2012). The chloroplast genome map of *V. albida* was drawn with OGDRAW (Greiner et al. 2019). The structure of the cis-splicing genes (Figure S1A) and the structure of the trans-splicing gene *rps12* (Figure S1B) in the chloroplast genome of *V. albida* were recognized using CPGview (Liu et al. 2023).

To show the phylogenetic position of *V. albida* in *Viola*, we analyzed 16 previously reported complete cp genomes in Violaceae, including *V. albida,* and an outgroup (*Passiflora foetida*; Passifloraceae). The selection of an outgroup for the phylogenetic analysis was based on a recent phylogenetic study (Xi et al. 2012). The genome sequences were aligned using MAFFT (v7.450; Katoh and Standley 2013) with the default option. For the Maximum-Likelihood (ML) analysis, the best model (General Time Reversible + G + I) was selected by the model test module included in the MEGAX (Kumar et al. 2018). For the ‘Gaps/Missing Data Treatment’, 95% of the site coverage cutoff was applied in both the model test and ML analysis. Five hundred bootstrap replications were applied to address node reliability in the ML tree.

## Results

**Figure 2.**
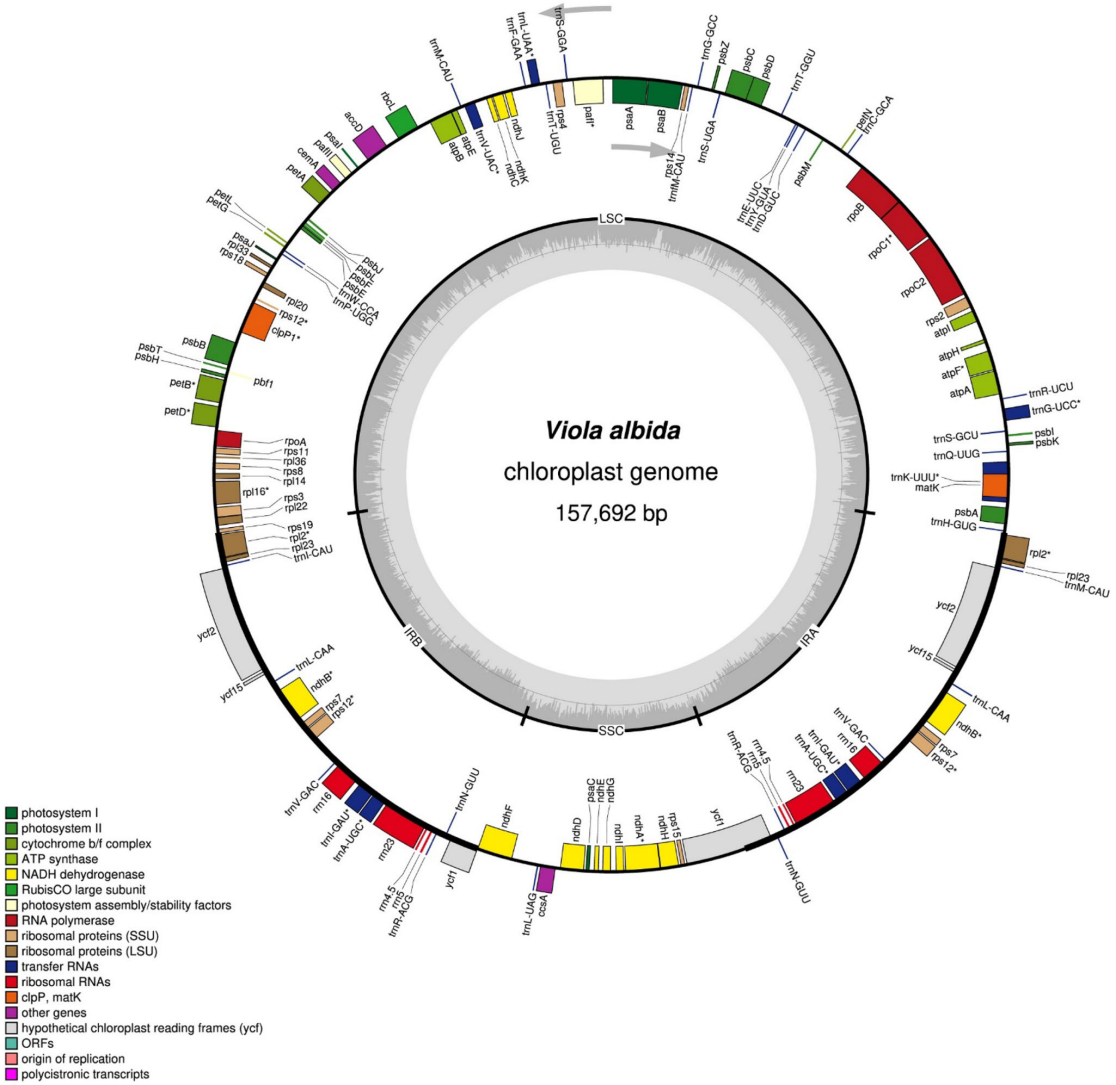
The circular chloroplast genome map of *V. albida* drawn by OGDRAW. Arrows indicate the direction of transcription of genes located inside and outside the large circle. The histogram inside the small circle shows the GC contents. Asterisk (*) indicates genes containing introns.

**Figure 3.**
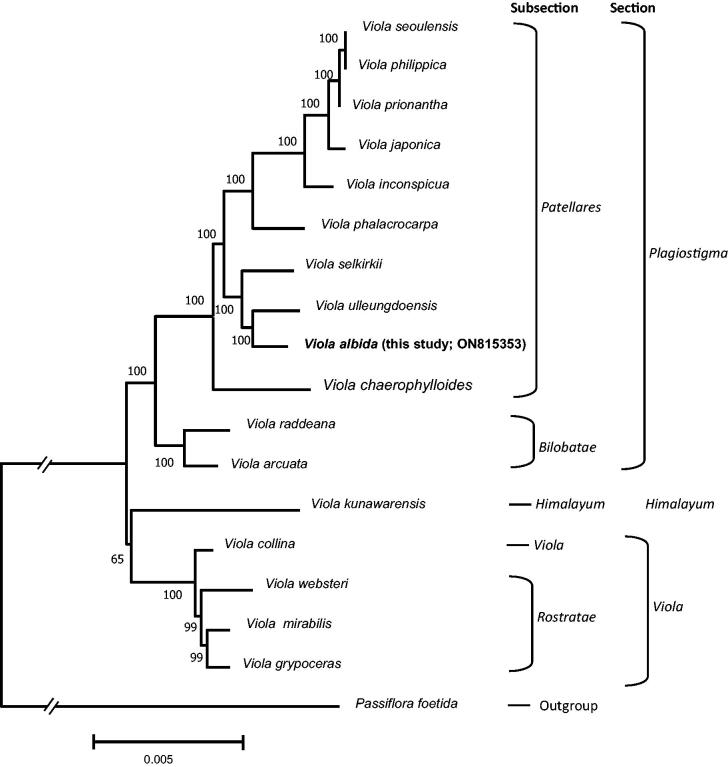
A tree generated from the maximum-likelihood analysis with 17 selected *Viola* cp genomes, including a genome determined in this study (*V. albida*) and an outgroup (*Passiflora foetida*). The number above the node indicates the bootstrap support with 500 replications. The following sequences were used: *V. seoulensis* NC_026986 (Cheon et al. 2017), *V. philippica* NC_052919 (Guo et al. 2021), *V. prionantha* NC_057663 (Duan et al. 2020), *V. japonica* NC_057486 (Cheon et al. 2020), *V. inconspicua* MW802532 (Cao et al. 2022), *V. phalacrocarpa* NC_041583 (Cheon et al. 2019), *V. selkirkii* NC_059909 (unpublished), *V. ulleungdoensis* NC_050744 (Yang et al. 2021), *V. albida* ON815353 (this study), *V. chaerophylloides* NC_065000 (Cao et al. 2022), *V. raddeana* NC_041584 (Cheon et al. 2019), *V. arcuata* NC_061694 (Moon and Kim 2022), *V. kunawarensis* NC_060866 (Zhou et al. 2022), *V. collina* MW802530 (Cao et al. 2022)*, V. websteri* NC_041585 (Cheon et al. 2019), *V. mirabilis* NC_041582 (Cheon et al. 2019), *V. grypoceras* NC_061911 (Park et al. 2023), and *Passiflora foetida* NC_043825 (Shrestha et al. 2019).

The cp genome sequence of *V. albida* was completed with an average coverage of 3,810.51 X (Figure S2). The complete cp genome of *V. albida* (GenBank accession number: ON815353) is 157,692 bp in length (36.3% of GC content) and consists of an LSC region of 86,220 bp, an SSC region of 17,248 bp, and a pair of IR regions of each 27,112 bp (Figure 2). There are 111 unique genes in the cp genome of *V. albida*, including 77 protein-coding genes, four rRNA genes, and 30 tRNA genes. The phylogenetic tree (Figure 3) shows that *V. albida,* of which the cp genome is determined in this study (ON815353), forms a clade with *V. ulleungdoensis,* and this clade is a sister to *V. selkirkii*. When we compare cp genome sequences between *V. albida* and *V. ulleungdoensis*, 647 substitutions, and 1,050 indels were identified. *V. chaerophylloides* is a sister to all other members of the subsection *Patellares*. The bootstrap analysis supports all of these relationships with high bootstrap values.

## Discussion and conclusion

When determining chloroplast genome sequences using mapping methods, we can minimize errors using the genetically closest genome. However, previous molecular phylogenetic studies of *Viola* didn’t provide information for the most relative genome to *V. albida* (Yoo and Jang 2010; Yang et al. 2021) because trees from these studies showed polytomy or low bootstrap supports. We, therefore, further mapped the raw reads against the three candidate cp genomes [*V. selkirkii* (NC_059909), *V. chaerophylloides* (NC_065000), and *V. phalacrocarpa* (NC_041583)], respectively. The resulting sequences are all identical, supporting the reliability of the final cp sequence from *V. albida*.

A previous phylogenetic analysis using eight chloroplast DNA regions showed that the two members of the VIOLA ALBIDA complex, *V. albida* and *V. chaerophylloides*, do not form a monophyletic clade (Yoo and Jang 2010). Although our analysis did not include *V. albida* var. *takahashii* because its cp genome is not published yet, our analysis based on complete cp genomes also confirmed this. However, since our phylogenetic tree shows only maternal inheritance, this does not entirely rule out the possibility that the species belonging to the VIOLA ALBIDA complex is a monophyletic group. To clarify the taxonomic structure of this complex, future studies should include intensive nuclear data analysis.

In this study, we report the cp genome sequence of *V. albida*. The results of our study will provide essential information for future intensive phylogenomic and evolutionary studies of the VIOLA ALBIDA complex as well as the genus *Viola*.

## Supplementary Material

Supplemental Material

## Data Availability

The genome sequence data supporting this study’s findings are openly available in GenBank of NCBI at https://www.ncbi.nlm.nih.gov/ under accession no. ON815353. The associated BioProject, SRA, and Bio-Sample numbers are PRJNA875227, SRR21363874, and SAMN30608856, respectively.
