# Supplementary material for: A complete chloroplast genome sequence of Viola albida Palibin 1899 (Violaceae), a member of VIOLA ALBIDA complex
Source: Mitochondrial DNA B Resour. 2023 Jun 18;8(6):673–7. doi: 10.1080/23802359.2023.2224462 (PMC10281375; doi:10.1080/23802359.2023.2224462)
Supplement: Supplemental Material [file TMDN_A_2224462_SM0753.docx]

**
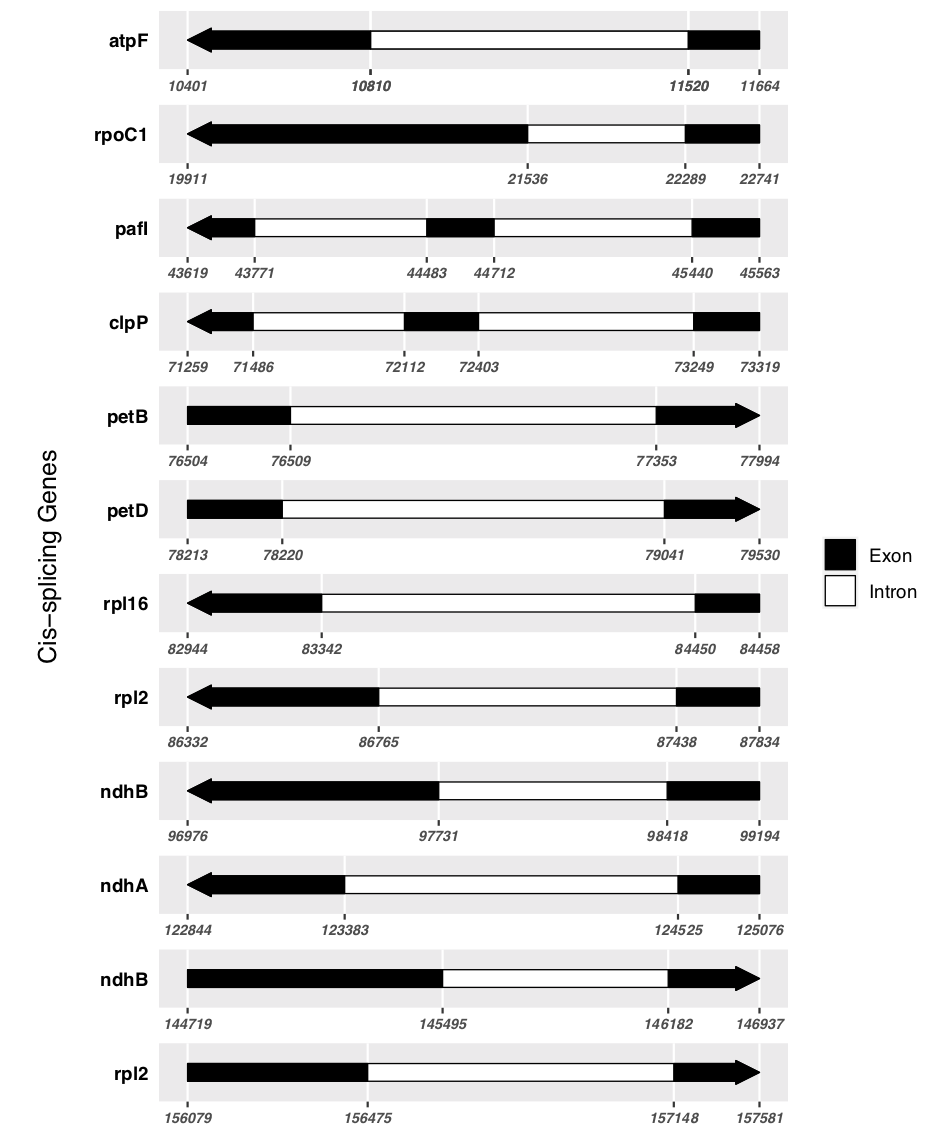
Supplementary material**

**B**

**A**


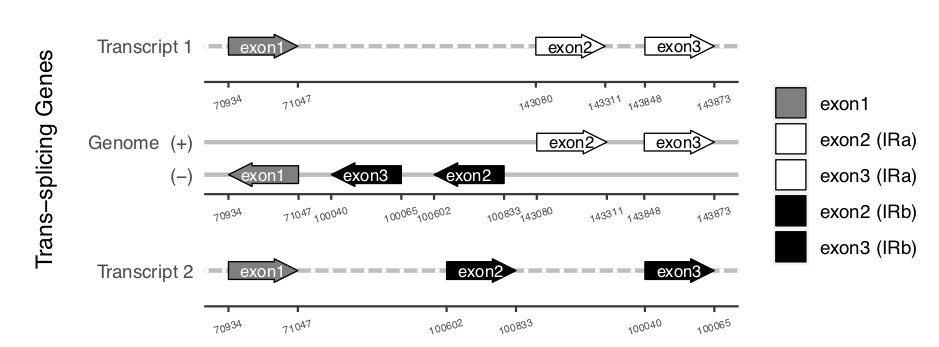


**Figure S1.** Schematic maps of (A) the cis-splicing genes and (B) the trans-splicing gene *rps12* in the chloroplast genome of *V. albida* (ON815353)*.*


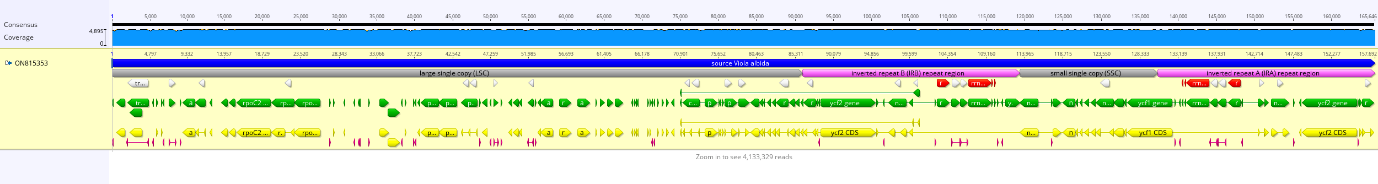


**Figure S2.** The coverage depth of the cp genome from *V. albida* (ON815353) with 4,133,329 paired-end reads. A light blue histogram shows the coverage depth. Dark blue bar, a reference sequence; gray and pink bars, four regions of the genome (LSC, SSC, and two IRs); short arrows, annotation types: red (rRNA), green (gene), yellow (CDS), and pink (tRNA).
